# ‘If It Was Easy Somebody Would Have Fixed It’: An Exploration of Loneliness and Social Isolation Amongst People Who Frequently Call Ambulance Services

**DOI:** 10.1111/hex.14167

**Published:** 2024-08-12

**Authors:** Lisa Moseley, Jason Scott, Gayle Fidler, Gina Agarwal, Cathy Clarke, Jonathan Hammond‐Williams, Carrie Ingram, Aidan McDonnell, Tracy Collins

**Affiliations:** ^1^ Faculty of Health and Life Sciences Northumbria University Newcastle upon Tyne UK; ^2^ North East Ambulance Service NHS Foundation Trust Newcastle upon Tyne UK; ^3^ Institute for Research on Aging McMaster University Hamilton Ontario Canada; ^4^ South Western Ambulance Service NHS Foundation Trust Exeter UK; ^5^ Northern Ireland Ambulance Service Health and Social Care Trust Belfast UK

**Keywords:** ambulance service, emergency medical services, frequent use, loneliness, social isolation

## Abstract

**Introduction:**

The aim of the study was to explore social isolation and loneliness in those who frequently contacted the ambulance service, what factors contributed to this and how unmet needs could be addressed.

**Methods:**

Semi‐structured interviews with staff from the ambulance service and service users who were identified as frequently contacting the ambulance service. Service users also completed the UCLA loneliness scale and personal community maps. Data were analysed thematically before triangulation with the UCLA loneliness scale and personal community maps.

**Results:**

The final analysis was drawn from 15 staff and seven service user participants. The relationship between social isolation and loneliness and contacting the ambulance service was a contributing, but not the driving, factor in contacting the ambulance service. For service users, we identified three key themes: (1) impact on activities of daily living and loneliness and/or isolation as a result of a health condition; (2) accessing appropriate health and social care services to meet needs; (3) the link between social isolation and/or loneliness and contact with the ambulance service. The analysis of staff data also highlighted three key themes: (1) social isolation and/or loneliness in their role; (2) access to other appropriate health and social care services; (3) the impact of austerity and Covid‐19 on social isolation and/or loneliness.

**Conclusions:**

Our research emphasises the complex nature of social isolation and loneliness, including the cyclic nature of poor health and social isolation and loneliness, and how this contributes to contact with the ambulance service.

**Patient or Public Contribution:**

The advisory group for the study was supported by a public and patient representative who contributed to the design of the study documentation, data analysis and authorship.

## 1 Introduction

People who frequently call ambulance services, defined in the United Kingdom as someone who calls the ambulance service five or more times in a month [1], do so because they often have unmet needs. This includes needs relating to physical health, mental health and/or social conditions and it is suggested that people are sometimes using emergency ambulance services as a means of last resort [2, 3]. People who call ambulance services (internationally referred to as emergency medical services [EMS]) frequently often make lower acuity calls and have increased contact during time periods when other services are unavailable [1, 4]. The number of people who frequently call each of the ten ambulance services in England averages around 300–500 per month (of which 100 are newly identified each month), though reporting of call volume measurement varies by service [1, 5]. Each ambulance service now has some form of proactive management of people who frequently call, such as clinical case management [6], which is currently being evaluated [7].

Amongst the social conditions that contribute to frequent use, social isolation and loneliness play a significant role [8], as well as being a wider contributor to non‐frequent use of ambulance services that may not be best placed to meet their needs and that are over‐capacity [9]. Further, a meta‐analysis of the impacts of social isolation and loneliness on health reported that the health impacts of social isolation and loneliness are not restricted to ambulance service use and impacts on higher uses of other health services, including emergency departments and higher avoidable admissions to hospital [10]. Social isolation and loneliness are distinct concepts; however, it is beneficial to consider both together due to their often interlinked and multifaceted nature [11]. Loneliness is a subjective emotional experience that can be felt even if the person is not alone [12, 13]. Numerous theories of loneliness exist, including those rooted in naturalism [14] and attachment theory [15], with a cognitive perspective presenting loneliness as a negative experience where there is a difference between desired and actual social relationships [16]. Recent studies have suggested that loneliness may be more complex and thus should include multiple other factors, such as identity and the person's perceived role in society [13]. Social isolation refers to the lack of social contacts; however, the quality of those contacts is also recognised as a factor in subjective feelings of social isolation [17]. Determinants of social isolation and loneliness include individual social and financial challenges and personal characteristics, as well as the size and composition of networks, which can fluctuate over time [18]. Social isolation and loneliness are linked to poorer physical health as well as mental well‐being, with loneliness and/or social isolation having an impact on health as well as existing health conditions being exacerbated by social isolation and/or loneliness [19, 20].

An understanding of the complexity of patient's lived experiences of loneliness and social isolation, including their unmet needs, can inform ambulance service and relevant community health and social care service responses to improve the quality of care and support for people who call ambulance services frequently. While each ambulance service now proactively manages people who call frequently there is currently no evidence that this is appropriate for social, rather than medical, concerns and work is developing to look at alternative models of care within the pre‐hospital care setting [21]. It is recognised that social and medical concerns are often overlapping in nature, for example, with mental health issues or substance use [22], or when a long‐term health condition contributes to social problems, such as social isolation and loneliness [23]. Little is known within ambulance services about how best to manage people who call frequently due to loneliness and social isolation, which is associated with reduced health and well‐being and may also be compounded by a loss of occupations, roles and relationships [24]. This was further exacerbated during the Covid‐19 pandemic, which saw a significant increase in social isolation and/or loneliness, the subsequent impacts on physical and mental health, leading to an increase in calls to the ambulance service [25, 26]. It is vital to develop appropriate responses and support for individuals experiencing social isolation and/or loneliness, thus potentially reducing the frequency of calls to ambulance services, which are currently over capacity.

The aim of this study was to explore the complex factors associated with loneliness and/or social isolation experienced by people who call ambulance services frequently.

## 2 Materials and Methods

### 2.1 Study Design

We utilised qualitative and quantitative methods, including personal community mapping (incorporating the development of a typology) [27], semi‐structured interviews and a loneliness measure (the UCLA 3‐Item Loneliness Scale), which is novel in this setting and with this population group. The personal community maps were utilised as a part of the interviews to guide discussion regarding social contacts and the UCLA 3‐Item Loneliness Scale was also completed during the interviews, which allowed for further exploration of loneliness. This mixed methods approach incorporated triangulation through the integration of data and allowed for a more comprehensive in‐depth exploration of the experiences and needs of this seldom‐heard, and potentially vulnerable, group that have not previously been examined.

The core study team was multidisciplinary, including an occupational therapist (principal investigator), a social worker (senior research assistant and interviewer), a psychologist and an ambulance service practitioner.

### 2.2 Participants

The study recruited service users who had contacted an ambulance service in England with staff recruited from the same ambulance service.

### 2.3 Recruitment

Service users were invited to participate via a letter, which was posted by the ambulance service. This letter was created in conjunction with the research advisory group, which included patient and public involvement (PPI). The ambulance service identified their most recent frequent callers (at the time of recruitment), excluded anyone where records indicated there would be concerns about capacity to consent or warning markers indicating that the person may pose a risk to the researchers, and contacted the first 100 service users after the exclusions. These service users were sent an invitation letter and an information sheet explaining the purpose of the study. Service users were asked to take part if they felt that social isolation or loneliness had contributed to their reason for contacting the ambulance service and to contact the researcher directly if they wished to participate. A reminder letter and the same information sheet were sent again after 2 weeks unless service users had contacted the research team to participate or asked not to be contacted further. The letters did not mention frequent use of the service. This process was repeated twice more with a total of 221 (males, *n* = 108; females, *n* = 113) service users contacted. The study aimed to recruit up to 20 service user participants.

Following the first 200 service users being contacted, the invitation letter was amended, with the input of the PPI representative, based on feedback received from those who had received letters and contacted the researcher to give feedback. The amended letter reiterated further that we were interested in speaking to people where social isolation or loneliness had been a factor in them contacting the ambulance service but, as the ambulance service does not routinely collect this data, this may not be applicable to them.

Staff were recruited via invitation emails to their work accounts and an intranet system. Potential participants were provided with a letter about the aim of the study as well as a detailed information sheet and asked to contact the research team directly if they wanted to participate. The study aimed to recruit a total of 15 staff, five from frontline paramedic roles, five from case management staff and five call handlers.

### 2.4 Data Collection

#### 2.4.1 Service User Interviews

**Figure 1:**
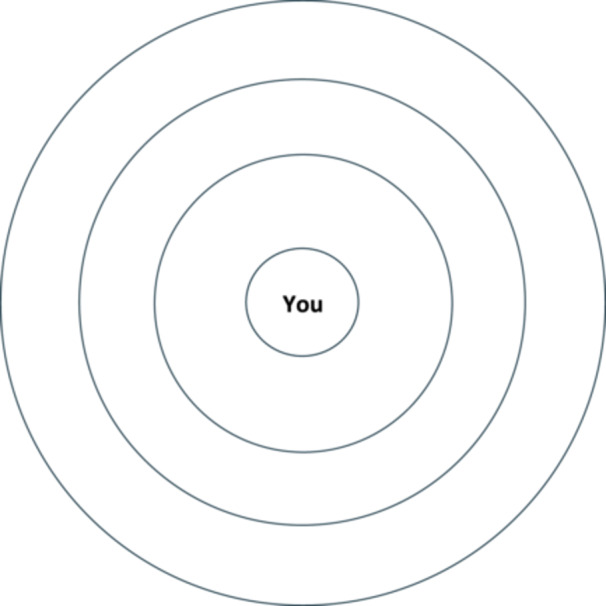
Personal community map. Inner circle—Name people who are very close and important to you. Middle and outer circles—Name people who are less close but still important to you.

**Figure 2:**
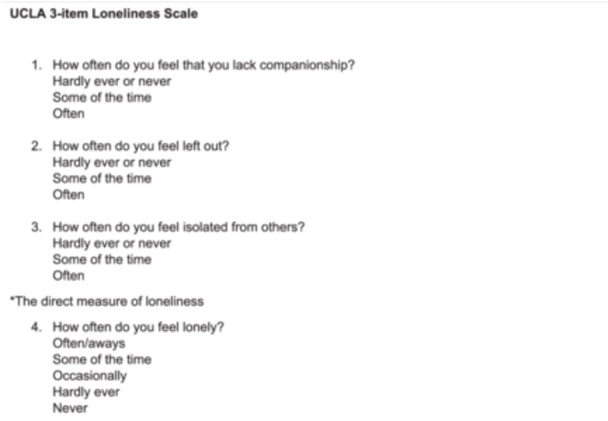
UCLA 3‐Item Loneliness Scale. *Hardly ever or never* = 1; *some of the time* = 2; *often* = 3. The lowest possible combined score on the loneliness scale is 3 (indicating less frequent loneliness) and the highest is 9 (indicating more frequent loneliness). *The individual responses to the UCLA questions may also be helpful in interpreting responses to the final question, ‘How often do you feel lonely?’. This question is not part of the UCLA scale and responses to this question should not be included in the UCLA combined score.

A topic guide was developed, with input from the research advisory group to explore people's social networks, their feelings of loneliness and social isolation, reasons for contacting the ambulance service and other services that could meet their needs, as well as information about their lives and experiences. Care was taken with the wording of the questions to ensure sensitivity and avoid the stigma associated with loneliness and social isolation. The interviews were recorded using an encrypted recorder and transcribed verbatim. Participants were given the choice to be interviewed face to face, via telephone or Microsoft Teams. As part of the interviews, personal community maps (Figure 1), based on those of Pahl and Spencer [28], and more recently Collins [27], were employed to identify social isolation and the structural components of the participants' social ties and contacts with family, friends, neighbours, the wider community and services. This hierarchical mapping technique uses simple diagrams consisting of three concentric circles. Participants were given information on the community mapping, asking them to name people they felt were close and important to them, with those in the inner circle being the closest to them and those in the outer two circles being less close but still important. The mapping technique does not impose any restrictions around how frequently someone may see that person, or have interaction with them, but the relationships and frequency of contact are explored as the community map is completed. The interviews also involved the completion of a short, standardised loneliness measure (UCLA 3‐Item Loneliness Scale) to determine participants' current levels of loneliness (Figure 2). A short measure is appropriate to prevent participant burden/fatigue during the interviews and has shown to be reliable and valid when compared to longer scales [29].

#### 2.4.2 Staff Interviews

A staff topic guide, developed with input from the wider advisory group, was used to explore the perceived prevalence of social isolation and loneliness in callers to the ambulance service, how social isolation and loneliness are identified, responses to social isolation and loneliness, the impact of the Covid‐19 pandemic and other services available for supporting service users with social isolation and loneliness. Interviews also collected information about the job roles of the participants, how long they had worked in the ambulance service and the geographical area they worked in.

Data collection took place between April and December 2023.

### 2.5 Analysis

Following verbatim transcription of the digital recordings of the interviews, data were analysed thematically to identify patterns and themes as well as contrasts [30]. Thematic analysis offers a practical and adaptable method for qualitative research data analysis. The team used NVivo Version 12 (Lumivero) [31], a qualitative data analysis software, to assist in the management and analysis of the data arising from the semi‐structured interviews. In the first instance, data from service user and staff interviews were analysed separately before triangulation in the final stage of coding.

Personal community maps were analysed for their content based on the number and composition of the participants' ties and contacts allowing for the development of a typology (Table 3). The number of people on the community maps was added to give a total number of contacts for each participant. The UCLA loneliness scales were also scored as the scale directs. The findings from the interview data, personal mapping data and data derived from the loneliness scale were triangulated. In addition, all members of the research team were involved in the analysis of the data to increase the trustworthiness of the findings. All participant names were pseudonymised.

## 3 Results

Of the 221 service users contacted, a total of seven interviews were conducted. Participant information is represented in Table 1. Of the participants who chose to take part, six received the original letter and one received the amended letter.

**Table 1:** Service user characteristics.

Participant name	Gender	Age	Marital status	Household composition	Main reported health concern
Fred	Male	79	Widowed	Lived alone	Reduced mobility
David	Male	87	Divorced	Lived alone	Cancer
Andrew	Male	75	Divorced	Lived alone	Chronic pain
Angela	Female	62	Single	Lived alone	Acquired brain injury and self‐injurious actions
Thomas	Male	65	Divorced	Lived alone	Chronic leg ulcers
Luke	Male	33	Single	Lived alone	Mental health and previous substance use
Joan	Female	77	Divorced	Lived alone	Arthritis, polymyalgia and reduced mobility

Fifteen staff members were recruited and details are represented in Table 2. Data were not available on the number of potential staff members who decided not to take part.

**Table 2:** Staff characteristics.

Participant name	Job role
Sally	Clinical section manager
Alison	Frequent caller officer
Caroline	Frequent caller officer
George	999 health advisor
Debbie	999/111 health advisor
Oliver	Clinical support team admin
Kerry	Senior health advisor
Louise	Paramedic and research paramedic
Michelle	Paramedic
Stephanie	Paramedic
Bethany	Paramedic
Anna	Clinical assessment service
Jane	Clinical assessment service
Claire	Clinical assessment service
Emily	Clinical assessment service

Service user interviews lasted between 19 and 74 min (mean = 47) and staff interviews lasted between 11 and 41 min (mean = 25).

### 3.1 Key Findings

#### 3.1.1 Service Users

The personal community maps and loneliness scale were analysed with a typology being attributed as shown in Table 3. The number of people included in the personal community maps ranged from 1 to 22. Most participants had family personal community types (four) and two had mixed types. The participants with the highest number of people in their diagram, Luke (12) and Joan (22) also had some of the highest loneliness scores, 9 and 6, demonstrating the complexity of loneliness and social isolation. This illustrates that people can still be lonely when they have people around them, it may also illustrate the importance of the perceived quality of relationships over quantity. Two participants had very restricted personal communities, with only one family member being included. All participants were either single, divorced or widowed and lived alone.

**Table 3:** Typology of personal communities.

Personal community type	Composition and size	UCLA‐3 scores
Family centred	Andrew—Only family in the diagram; family in the inner circle 1 member	Andrew—3
	Angela—Only family in the diagram; family in the inner circle 1 member	Angela—7
Family with mixed centrality	Fred—Majority family in the diagram; family and friends in the inner circle 5 members	9
Concentrated family	Luke—Majority family in the diagram; family in the inner circle 12 members	9
Mixed with family centrality	Thomas—Equal number of family and friends in the diagram; family in the inner circle 2 members	4
Mixed with mixed centrality	David—Equal number of family and friends in the diagram; family and friends in the inner circle 4 members	David—3
	Joan—More family than friends in the diagram; equal mix of family and friends in the inner circle 22 members	Joan—6

The relationship between social isolation and/or loneliness and contact with the ambulance service was complex. The service user participants were asked to self‐identify if they were socially isolated and/or lonely and if this had impacted their contact with the ambulance service, but during the interviews, it was difficult for them to articulate this relationship. Despite this difficulty, social isolation and/or loneliness were both recognised to be exacerbated by a health condition that may have contributed in some form to contact with the ambulance service, albeit not as a necessary nor sufficient factor. The analysis generated three key themes relating to this relationship: (1) impact on activities of daily living and loneliness and/or isolation as a result of a health condition; (2) accessing appropriate health and social care services to meet needs; (3) the link between social isolation and/or loneliness and contact with the ambulance service.

#### 3.1.2 Impact on Activities of Daily Living and Loneliness and/or Isolation as a Result of a Health Condition

All participants interviewed identified that a current health condition was impacting their ability to undertake day‐to‐day activities, which included leaving the house, and the significant impact this was having. Fred, who has reduced mobility and was awaiting a knee replacement, summed up the impact:

… [I] wander round … I see how I can cope … but I'm back within an hour, back on my own …

(Fred, male, age 79)

Not being able to undertake day‐to‐day activities and spending long periods alone impacted participants. Luke, who had been experiencing panic attacks, described this:

I'm here like 24/7 with just me and my thoughts.

(Luke, male, age 33)

Similarly, Joan also discussed how being alone was impacting her well‐being:

… I've felt … anxious being on my own … the district nurse … spoke to [the social worker], and she said I think the problem here is … she's frightened to be on her own … I am.

(Joan, female, age 77)

Alongside the impact of traditionally defined social isolation and/or loneliness, we also found a more complex picture of social isolation and loneliness. David and Andrew reported the lowest number of social contacts on their personal community maps while scoring as least lonely on the UCLA scale. They described themselves as naturally enjoying their own company and not wanting to seek out social contact. However, they also described a profound sense of loss and purpose due to losing a business and from no longer being able to partake in a hobby. In both instances, the service user described how their declining health had meant they had no other choice but to cease these activities. Andrew, a 75‐year‐old man who has chronic pain following a prostate biopsy, summed this up:

I don't feel left out I just … want my life back, I want to get back to doing what I was doing. I was fully occupied … I'm just quite happy to get on with my own life, do my do my own thing but that … hospital examination … destroyed it all.

(Andrew, male, age 75)

While service users interviewed may not have been impacted directly by social isolation and/or loneliness, there was a different type of loneliness linked with a loss of occupational activity. Alongside social activities and hobbies that service users had previously enjoyed, they also discussed the impact of the loss of smaller everyday activities, such as going shopping, visiting the local town or even maintaining the garden where they would see neighbours. These activities, while not necessarily defined as social activities, provide opportunities for social interaction and conversation, if only briefly, and the inability to continue with these activities contributed to the loneliness felt by participants. Fred stated:

I'm frightened … I'll not go out, if I've got to … I'm really chuffed when I get there and back … like today it was nice going to Sainsbury's … having something to eat and just seeing people.

(Fred, male, age 79)

#### 3.1.3 Accessing Appropriate Health and Social Care Services to Meet Needs

Access to primary and secondary health services, as well as social care services, was a contributing factor for calls to the ambulance service. There was no identified direct link between social isolation and/or loneliness and the impact of accessing health and social care services. However, there was a more complex relationship where participants expressed their health and social care needs were not being met, which impacted their current health and their ability to undertake day‐to‐day activities, and this subsequently contributed to their perceived social isolation and/or loneliness. Four of the service user interviews highlighted the difficulties in arranging an appointment with their general practitioner (GP), getting referrals to services and in two instances service users had been removed from their GP practice. Andrew advised that his GP had removed him due to a breakdown in the relationship and that he now had to travel much further to a GP practice; however, Thomas did not want to give any further details. Fred stated, when discussing access to his GP, that:

I've got a better chance of seeing the Pope, you know, you just can't get [appointments].

(Fred, male, age 79)

Two participants also discussed the physical difficulties in accessing their GP due to location and that attempts to move to other practices had been unsuccessful. David, who had been having panic attacks since carbon monoxide poisoning as well as cancer, described his difficulties:

… my … doctor's surgery is in the centre of [city] and it's the top of a shopping mall … I've tried to change my doctors … they've put things in operation for me to move … and it … just hasn't happened.

(David, male, age 87)

Similarly, participants discussed the difficulties in accessing social care and community services that may reduce feelings of social isolation and loneliness. Fred reported that he had attempted to engage with some social groups; however, one had closed, one had a very long waiting list and he did not feel one was appropriate for him, which highlights not just the need for services but varying services to meet different needs:

… [a] couple of months ago I went to this friendship club, I walked in the door and my heart sank. There was about eighty old ladies … and they were going to play bingo and that was not me.

(Fred, male, age 79)

Social isolation and loneliness do not just stem from lack of services but also the ability to engage in an occupation, which we've termed ‘occupational loneliness’. Addressing the barriers to these occupations may reduce occupational loneliness. Andrew talked about not wanting to socialise but to return to his activities:

I just went my own … way with my hobbies and interests … . I'm not one the lads, I'm just me … I'd rather sit on a railway station than sit in a working man's club.

(Andrew, male, age 75)

The youngest participant, Luke, had recently successfully finished a methadone programme but was experiencing panic attacks, which led to contact with the ambulance service. Luke did not know of any services, and had not been referred to any following the methadone programme, that could support him until speaking with the research interviewer who was able to signpost:

No … they just stop following you up. Once you come off the methadone … that's their case done with you … I was surprised … because when you're coming off … you're at high risk of relapsing … I don't know what services are … it's hard … you get referred onto them by other people.

(Luke, male, age 33)

There was also dissatisfaction with formal care services. Angela, who had an acquired brain injury, frequent falls and self‐injurious actions, reported a high level of mistrust in her social worker:

… with the social worker … they are more dangerous and harmful to me … .

(Angela, female, age 62)

Thomas, who had chronic leg ulcers that impacted his mobility, was equally dissatisfied with the support he was receiving from social workers and felt that they were not meeting his needs:

I don't ring them … [my social worker is] just not interested.

(Thomas, male, age 65)

Fred and David had both had carer support put in place; however, neither felt that the support that was provided, carer visits multiple times a day, was meeting their needs. David commented:

I had a [social worker] here who … came to see what my needs were … I didn't need these people coming because they … were just interrupting my lifestyle …

(David, male, age 87)

Joan also had carer support coming in but felt it was not meeting her needs and that they were not respecting her home:

… I went mad, I said ‘you're upsetting my routine’. You see this them care workers must have put them in there, they must have just gone like that [referring to paperwork and small belongings that had been placed in a fruit bowl haphazardly].

(Joan, female, age 77)

#### 3.1.4 The Link Between Social Isolation and/or Loneliness and Contact With the Ambulance Service

These factors culminated in contact with the ambulance service. David discussed that he had been contacting the ambulance service due to panic attacks that started after he had carbon monoxide poisoning:

I called a lot of 999s [emergency care number] and 111s [urgent care number] because I would get myself into such a state … I would panic like mad … still couldn't settle myself down and … eventually I would ring.

(David, male, age 87)

Andrew shared that he had contacted the ambulance service not only because of the physical pain he was experiencing but also due to the anger he felt about his physical symptoms. Andrew discussed that he felt that no one was managing his symptoms or understanding the significant impact this was having on his life:

I … [rang because of] … physical [symptoms] and a lot of it is anger, anger and what has been done to me and I start thinking about the way I've been left to suffer …

(Andrew, male, age 75)

Fred explained a different experience that led to calls to the ambulance service. While he had called the ambulance service himself on a few occasions, the ambulance mostly attended due to his son ringing him. Fred described himself as fiercely independent and that he did not want support to undertake household tasks but that his issues with his mobility meant that he frequently fell. Fred's son lived abroad, and Fred had no family or friends nearby able to support him, so when his son could not contact Fred he would contact the ambulance service:

There's a lot of times he's phoned me up, on the landline, and he doesn't get me on the mobile there's about five or six times he's called the ambulances out

(Fred, male, age 79)

### 3.2 Staff

Staff interviews supported the complicated interaction between social isolation and/or loneliness and how this linked to people contacting the ambulance service. Analysis of staff data generated three key themes: (1) managing service user social isolation and/or loneliness in the ambulance service; (2) access to other appropriate health and social care services; (3) the impact of austerity and Covid‐19 on social isolation and/or loneliness.

#### 3.2.1 Managing Service User Social Isolation and/or Loneliness in the Ambulance Service

Staff interviews highlighted that more service users were experiencing social isolation and/or loneliness and managing this was becoming more prominent in their role. This was summed up by Louise:

… the demographic of who we go to from when I started, say fifteen plus years ago … is completely different … it used to be a lot more … patients who … were either seriously unwell or injured … I think as time gone on the ambulance service has come become a one stop shop for everything. There's quite a lot of … patients referred to us because other services … say they're not going to get involved.

(Louise, paramedic and research paramedic)

Stephanie highlighted the interlinked nature of social isolation and/or loneliness, and that while the person has phoned for a health concern, there are other factors impacting this:

You've done the formal physical assessment, you've done the formal history taking … but it's the other bits that come out just in chat … the thing you've told us about, the top level thing … . is what's going on but again it's fed by all of this underlying stuff as well.

(Stephanie, paramedic)

Staff tended to initially attribute social isolation and loneliness to the older population but quickly reflected and discussed social isolation and loneliness impacting people throughout the life course, with Claire highlighting that it was:

… spread across all ages. I do find sometimes the older generation … will just call for a chat but it can be any age.

(Claire, clinical assessment service)

The staff's understanding, and recognition of social isolation and loneliness, highlighted a lack of knowledge regarding the complexity of the concepts. This included references to people not being lonely because they had some social contact, that living in residential or supported environments meant that people would not be socially isolated or identifying them as lonely or isolated because they lived alone. For example, Anna, initially spoke of a service user to illustrate someone who was lonely or socially isolated but then discounted this assessment because she had some family visits and was able to leave her house:

… I think … she just wanted someone to talk to and yet I don't think she was necessarily lonely because a daughter came, her son came … she wasn't house bound or anything like that …

(Anna, clinical assessment service)

However, staff who only had contact with service users over the phone did recognise that it was difficult for them to identify social isolation and loneliness, felt this was beyond the remit of their role and that they often did not have time to explore issues. This was explained by Debbie:

… generally I think when you're going through your assessment it's not necessarily something you're going to pick up on.

(Debbie, 999/111 health advisor)

Staff who visited service users in their homes highlighted that the physical living environments of those they perceived as socially isolated and/or lonely were often sparse and that the service users themselves were also unkempt, as highlighted by Louise and Stephanie:

… you can tell that people don't see anybody, sometimes they can live in quite sort of stark environments … They're not … homely or cosy …

(Louise, paramedic and research paramedic)

they're not very well kempt … you know like poor hygiene …

(Stephanie, paramedic)

Further staff who attended service users in person felt that this made it easier to recognise social isolation and/or loneliness as highlighted by Louise:

… it will come about through what they say … we get the … added benefit I suppose in terms of assessing somebody when we see them in their own home environment

(Louise, paramedic and research paramedic)

Every staff participant interviewed advised that they received no training in relation to social isolation or loneliness, which may have contributed to how they perceived social isolation and loneliness.

#### 3.2.2 Access to Other Appropriate Health and Social Care Services

Staff felt that calls to the ambulance service had increased due to difficulties in GP access and long waiting times for other services, as well as less frequent contact with their GP, which was seen to increase social isolation and/or loneliness and it was often the only social contact that some people had. Caroline discussed both these factors:

That's the one that comes up quite often … because they can't get in touch with their GP … trying to get any sort of contact with the GP … I would say a lot of my frequent callers … like to have that contact with the GP … a little bit old fashioned where they think the GP has the answer to everything. I just think they like to have that that contact with people.

(Caroline, frequent caller officer)

Concerns around access to mental health services leading to people phoning the ambulance more frequently were also noted in 13 out of 15 staff interviews. Alison highlighted how complex this can be and that mental health, social isolation and loneliness, as well as other conditions, can be interlinked:

You might see predominately one thing but when you look into it a little bit further, it's like … a vicious circle and … it might start with mental health, alcohol, but it might also be social isolation, loneliness … it can all be like interweaved.

(Alison, frequent caller officer)

Access to social care services was also discussed by staff as a factor in the increase in calls where social isolation and/or loneliness were a factor. Some staff worked within the team that manages people who frequently call the ambulance service and had a wider range of options for referrals; however, those services were for people who contacted the ambulance or health services frequently. Staff also spoke about services that had historically been available to people, such as day centres and community groups, which have been slowly eroded due to budget constraints and more recently the Covid‐19 pandemic. Sally discussed the change she had noticed over the years of working in the ambulance service:

… years ago you used to have lots of day care centres, there was lots of places, lots of activities for these people to do … they don't seem to exist anymore.

(Sally, clinical section manager)

#### 3.2.3 The Impact of Austerity and Covid‐19 on Social Isolation and/or Loneliness

Staff interviews highlighted that the societal challenges following Covid‐19 and the ongoing impacts of austerity have contributed to increased levels of social isolation and loneliness. Of the 15 staff interviewed, 13 highlighted Covid‐19 as a factor that remained ongoing at the time of interviews, as discussed by Michelle:

a lot of people did really struggle during the pandemic and during isolation … I think people then started to suffer with more mental health problems … due to the isolation and that's still ongoing …

(Michelle, paramedic)

Bethany shared a particularly difficult story illustrating the impact of loneliness after she had responded to a medication overdose of an elderly lady due to isolation during the pandemic:

… found her … unresponsive on the bed but breathing, with a little note saying … I can't take this anymore … unfortunately … she did end up passing away in hospital later on.

(Bethany, paramedic)

Ongoing austerity was felt to be impacting a wide range of services, which five staff members commented was impacting the ambulance service. In relation to social isolation and loneliness, Alison commented on the erosion of community services:

… it's all down to money, there's not a lot of them kind of services out there anymore.

(Alison, frequent caller officer)

There were also comments on the impact on staffing and services, which was contributing to delayed responses from other services highlighted as integral in supporting loneliness and/or social isolation. Anna highlighted:

… like us everywhere erm are understaffed and overworked.

(Anna, clinical assessment service)

## 4 Discussion

The aim of this study was to explore the factors associated with loneliness and social isolation experienced by people who call ambulance services frequently. The findings illustrate a complex and interconnected narrative of social isolation and loneliness and how this is a linked, but not the driving factor, in ambulance service use. While current theories on loneliness differ, they centre around relationships [11, 14, 15, 16], which we argue does not fully encompass ‘occupational loneliness’, as evident in our interviews with service users. When discussing loneliness, participants did not describe feelings of loneliness associated with a lack of social or unsatisfactory quality of existing relationships, as would be traditionally considered as loneliness, nor did they score highly on the loneliness scale. Instead, their loss of activities manifested in comparable ways to those who did score highly on the loneliness scale. Further, all service user participants reported using the ambulance for health reasons but recounted the factors that compounded social isolation and loneliness, the loss of hobbies and the absence of everyday ‘micro‐interactions’ usually achieved in the course of everyday life through activities such as shopping. These stemmed from health issues and further exacerbated health concerns due to the impact of social isolation and loneliness on mental well‐being, supporting findings of previous research [19, 20]. We contend that current definitions of social isolation and loneliness need to widen to include occupational loneliness and the loss of micro‐interactions.

**Figure 3:**
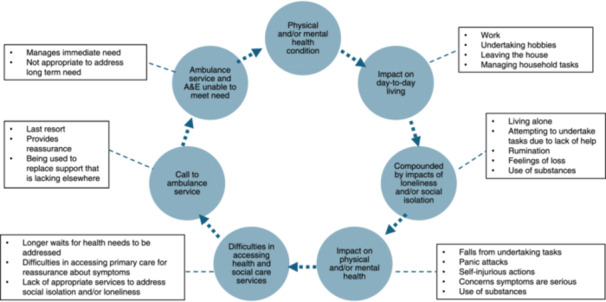
Relationship between social isolation and/or loneliness and contact with the ambulance service. Dashed lines represent relationships that are not necessary nor sufficient for people frequently contacting the ambulance service and are part of a complex multifactorial system.

While social isolation and/or loneliness were not the sole factors in contacting the ambulance service, their linked nature means that it is still having an impact. In epidemiological terms, our study suggests that social isolation and loneliness are neither necessary nor sufficient for people frequently contacting the ambulance service, and is therefore part of a complex multifactorial relationship (see Figure 3 for a demonstration of this relationship). Exploration of the use of ambulance services for conditions that could be treated within primary care has highlighted that isolation, along with feelings of being overwhelmed, and alternative avenues of care not being available all contribute to the decision to contact the ambulance service [9]. This reflects a wider change in the role of ambulance services, which are receiving increased calls for social needs and conditions that could be dealt with in primary care [32]. Booker et al. [9] found that service users value not only the medical care they are given but also reassurance and support in overwhelming circumstances and active management of a situation.

Ambulance service staff increasingly provide holistic, person‐centred care, which requires adequate training to support the identification of social issues and onward referral to appropriate services [33]. We identified that the complexity of social isolation and/or loneliness was not always fully understood by staff. While some staff interviews highlighted the very serious potential impacts of social isolation and loneliness, such as subsequent mental health concerns and physical health issues, some of the staff felt that it was difficult to recognise social isolation or loneliness. As such staff made hasty judgements on simple measures such as whether someone lived alone and that this was not something that they would routinely discuss within their role. Findings suggest that social isolation and loneliness are likely more prevalent than previously recognised [34] with a recent study finding more than one in 10 adults reporting social isolation [35]. All staff participants responded that they had had no training in relation to social isolation and loneliness but would welcome this. Future research should also consider the ambulance service role in meeting these needs and whether they can provide interventions beyond signposting, as part of the changing role of ambulance service staff.

This research also highlights that the multifactorial relationship between social isolation and loneliness and the use of the ambulance service includes poor access to other services, and therefore needs to be addressed at a wider system level. Loneliness and social isolation are clearly identified as eligible needs under the Care Act 2014 [36], with local authorities having a duty to meet these needs where they have a detrimental impact on people's well‐being, as well as having a duty to reduce, prevent and delay these needs from arising. The service user interviews highlight that the support they received focused on the practical elements of daily living and not their wider social needs. However, this should be considered in the context of austerity and the difficult choices that local authorities have to make in the face of budget cuts. As such, it is difficult to recommend that local authorities ensure they are considering these needs when assessing individuals without also calling on the government to properly fund the required services. A review showed that in the period between 2010 and 2018, there had been a 31% reduction in spending on social care and in that period there was a substantial increase in hospital visits by those over 65, of which the authors concluded half were directly attributable to the cuts in long‐term care spending [37]. The majority of attenders in this category had no diagnosable medical concern as a conclusion of their visit [37]. While this examined emergency department use, given the evidence from our interviews, this link should also be further explored in the context of ambulance service use.

Given the findings from this study and prior studies, the wide repercussions of social isolation and/or loneliness, as well as the complexity of service use, we make a further recommendation that specific training on recognising and responding to social isolation and/or loneliness is made a priority across health and social care services as well as formulating a response to tackle social isolation and/or loneliness. The evidence on the causal mechanisms of loneliness and/or social isolation is clear [38] and as such should be prioritised as a public health concern.

The majority of service users who volunteered to take part in the study were men. These participants were predominately older and previous research suggests that older males tend to have fewer social contacts and are more likely to be lonely [27], with older males reporting being lonely at double the rate of the general population [35]. As such, the authors considered given that the subject matter was more likely to impact males this may have been a contributing factor to their participation. One of the younger participants, Luke, also stated that as a man he felt that men did not really show their emotions and that this research had been his opportunity to discuss this and highlighted that this was the first time he had shared his full story and feelings with anyone. This fits with the discourse of the masculine identity and that men can find it more difficult to admit, discuss or seek help for issues that they feel undermine this masculinity [39]. This further illustrates how gender influences experiences of loneliness and social isolation and that consideration needs to be given to supporting men, with an understanding of the factors that may influence men in seeking out and accepting support [40].

While the study was limited to one ambulance service, results are transferable, and the area is socioeconomically diverse. We asked service users to self‐select where they felt social isolation and/or loneliness may have been a factor in their contact with the ambulance service. As such it is recognised that service users who did not realise that social isolation and/or loneliness may be a factor, or did not feel comfortable admitting this, would not have participated and may have contributed to low recruitment numbers. Further research should examine how to better identify social isolation and/or loneliness in people who are frequently using health services, as well as how best to engage and recruit them in research.

## 5 Conclusions

As the first study to specifically explore the impact of social isolation and/or loneliness in relation to people frequently using the ambulance, we offer a novel insight into the complex relationship, which is exacerbated by existing health conditions and thus creating a cyclic impact. The research also highlights the changing role of ambulance services in responding to social needs or where health needs have been exacerbated as a result of social needs. Ambulance services and other services with responsibility for people's care should recognise the multifactorial nature of how social isolation and/or loneliness contribute to the use of healthcare services. In doing so, training should be implemented to support health and social care staff in recognising and responding to people where social isolation and/or loneliness are factors in their service use. The wider discourse on social isolation and loneliness should also consider the loss of micro‐interactions associated with occupation instead of just focussing on a lack of social contacts or networks. Future research should examine how the ambulance service can respond to these needs as well as the wider systematic changes required to address the factors contributing to social isolation and loneliness.

## Author Contributions

**Lisa Moseley:** data curation, formal analysis, investigation, project administration, resources, methodology, visualisation, writing–original draft, writing–review and editing. **Jason Scott:** conceptualisation, data curation, formal analysis, funding acquisition, methodology, resources, project administration, supervision, validation, visualisation, writing–original draft, writing–review and editing. **Gayle Fidler:** conceptualisation, funding acquisition, methodology, resources, validation, visualisation, writing–review and editing. **Gina Agarwal:** methodology, validation, writing–review and editing. **Cathy Clarke:** validation, methodology, visualisation, writing–review and editing. **Jonathan Hammond‐Williams:** methodology, validation, visualisation, writing–review and editing. **Carrie Ingram:** methodology, validation, visualisation, writing–review and editing. **Aidan McDonnell:** methodology, validation, visualisation, writing–review and editing. **Tracy Collins:** conceptualisation, data curation, formal analysis, funding acquisition, methodology, project administration, resources, supervision, validation, visualisation, writing–original draft, writing–review and editing.

## Ethics Statement

This study received ethical approval from Northumbria University (No. 01947) and the Health Research Authority (Nos. 321213 and 323064).

## Conflicts of Interest

The authors declare no conflicts of interest.

## Data Availability

The data generated during this study are not publicly available as participants did not give consent for data sharing. The authors would be happy to interrogate the data on behalf of others upon reasonable request and subject to necessary ethical approvals.
